# Improved Autophagic Flux in Escapers from Doxorubicin-Induced Senescence/Polyploidy of Breast Cancer Cells

**DOI:** 10.3390/ijms21176084

**Published:** 2020-08-24

**Authors:** Agnieszka Bojko, Karolina Staniak, Joanna Czarnecka-Herok, Piotr Sunderland, Magdalena Dudkowska, Małgorzata Alicja Śliwińska, Kristine Salmina, Ewa Sikora

**Affiliations:** 1 Laboratory of Molecular Bases of Aging, Nencki Institute of Experimental Biology, Polish Academy of Sciences, 3 Pasteura St., 02-093 Warsaw, Poland; a.bojko@nencki.edu.pl (A.B.); k.kucharewicz@nencki.edu.pl (K.S.); j.czarnecka@nencki.edu.pl (J.C.-H.); p.sunderland@nencki.edu.pl (P.S.); m.dudkowska@nencki.edu.pl (M.D.); 2 Laboratory of Imaging Tissue Structure and Function, Nencki Institute of Experimental Biology, Polish Academy of Sciences, 3 Pasteura St., 02-093 Warsaw, Poland; m.sliwinska@nencki.edu.pl; 3 Cancer Research Division, Latvian Biomedical Research and Study Centre, LV-1067 Riga, Latvia; salmina.kristine@gmail.com

**Keywords:** autophagy, autophagic index, cancer, DNA damage, SQSTM1/p62, polyploidy, senescence, senescence escape, TFEB, Rubicon

## Abstract

The induction of senescence/polyploidization and their role in cancer recurrence is still a poorly explored issue. We showed that MDA-MB-231 and MCF-7 breast cancer cells underwent reversible senescence/polyploidization upon pulse treatment with doxorubicin (dox). Subsequently, senescent/polyploid cells produced progeny (escapers) that possessed the same amount of DNA as parental cells. In a dox-induced senescence/polyploidization state, the accumulation of autophagy protein markers, such as LC3B II and p62/SQSTM1, was observed. However, the senescent cells were characterized by a very low rate of new autophagosome formation and degradation, estimated by autophagic index. In contrast to senescent cells, escapers had a substantially increased autophagic index and transcription factor EB activation, but a decreased level of an autophagy inhibitor, Rubicon, and autophagic vesicles with non-degraded cargo. These results strongly suggested that autophagy in escapers was improved, especially in MDA-MB-231 cells. The escapers of both cell lines were also susceptible to dox-induced senescence. However, MDA-MB-231 cells which escaped from senescence were characterized by a lower number of γH2AX foci and a different pattern of interleukin synthesis than senescent cells. Thus, our studies showed that breast cancer cells can undergo senescence uncoupled from autophagy status, but autophagic flux resumption may be indispensable in cancer cell escape from senescence/polyploidy.

## 1. Introduction

Despite the spectacular progress in cancer treatment made during recent years, some types of aggressive cancer are still able to spread easily and become resistant to anticancer treatment. One of the reasons for this could be the phenomenon of therapy-induced senescence (TIS).

TIS, which halts cancer cell proliferation instead of inducing cell death, became a desirable outcome of cancer treatment [1,2]. However, it eventually turned out that the senescence of cancer cells can have an adverse effect of radio/chemotherapy. Indeed, senescent cells secrete many factors that modify the microenvironment, which in turn favor cancer development [3]. Moreover, the senescence of cancer cells can be reversible. Interestingly, it seems that the reversibility of cancer cell proliferation arrest is associated with their therapy-induced polyploidization [4,5,6,7,8,9,10,11,12,13]. The progeny of polyploid senescent cells regain the ability to proliferate, together with depolyploidization. Thus, TIS could represent a mechanism of evasion from the toxicity of chemotherapy and radiation, facilitating cancer recurrence [14].

Senescent cancer cells, beside division arrest and secretory activity, known as the senescence-associated secretory phenotype (SASP), are, similarly to senescent normal cells, characterized by many other features, such as the increased activity of senescence-associated β-galactosidase (SA-β-gal), lipofuscin and lipid droplet accumulation, altered morphology (flattening and increased granularity), an increased level of cyclin-dependent kinase inhibitors, such as p16INK4A and p21WAF1/CIP1 [15], increased lysosomal mass [16], morphologically and functionally altered mitochondria [17] and DNA double-strand breaks (DSBs). The latter induces the so-called DNA damage response (DDR) signaling pathway [18]. The most frequently analyzed key proteins of the DDR, which include DNA DSB sensors, mediators and executors, are: γH2AX, 53BP1, p-ATM, p-ATR, p-p53 and p-CHK2 [6]. Cellular senescence, characterized by increased metabolism, is closely interrelated with autophagy, however, senescence may be a result of autophagy impairment or, on the contrary, senescence may lead to autophagy dysfunction. Autophagy in senescent cells, especially cancer senescent cells, is highly dependent on the cell type and context [19]. It has been postulated by Erenpreisa et al. [20] that transiently senescent cancer cells acquire additional DNA repair capacity through mitotic slippage and entering a sequence of ploidy cycles, which facilitate the repair and sorting of damaged DNA, ultimately promoting the genesis of mitotically competent daughter cells following final depolyploidization. It has been stated that autophagy is required to fuel this process [21].

Autophagy is a catabolic process in which macromolecules and organelles are degraded and recycled, thus providing metabolites to maintain the energy supply in the cell. A characteristic feature of macroautophagy (herein referred to as autophagy) is the formation of vesicles, called autophagosomes, that enclose the degradation-bound cargo and, subsequently, fuse with lysosomes, giving rise to autolysosomes, wherein the cargo is degraded and recycled. The formation of the autophagosome includes phagophore nucleation, elongation and vesicle completion, which are tightly regulated by various autophagy-related proteins, e.g., the ULK1/2 complex, the class III PtdIns3K complex and LC3-II [22]. Autophagy is regulated at each stage, i.e., initiation, vesicle fusion or cargo degradation, by external factors or by endogenous modulators, e.g., Rubicon, the protein present in the Beclin complex, and inhibiting autophagy [16]. As autophagy is a highly dynamic process, for proper estimation of autophagy efficiency, it is crucial to measure the autophagic flux, which determines the degradation activity [22].

Although the modulation of autophagy is a very important part of cancer therapy [23], no therapies are currently available that specifically focus on autophagy modulation. Moreover, there are many controversies in the literature concerning the role of autophagy in cancer cell senescence and, as has been pointed out [24], it is difficult to judge whether and how these two processes are interconnected.

Accordingly, in this study, we aimed to answer the question about autophagy modulation in breast cancer cells induced to senescence following doxorubicin treatment. We chose MDA-MB-231 and MCF-7 cells. MDA-MB-231 cells are triple negative breast cancer and have a mutated form of p53 [25,26], whereas MCF-7 cells possess an estrogen receptor and WT p53 [26,27]. Moreover MDA-MB-231 cells, in contrast to MCF-7, have a very low basal autophagy level [28]. Despite this, we expected the senescence process in both types of cells to be connected with polyploidization and senescence escape [29]. Thus, we were interested in propensity to undergo senescence and autophagy activity in the escapers.

## 2. Results

### 2.1. Doxorubicin-Induced Senescence of MDA-MB-231 Cells

**Figure 1:**
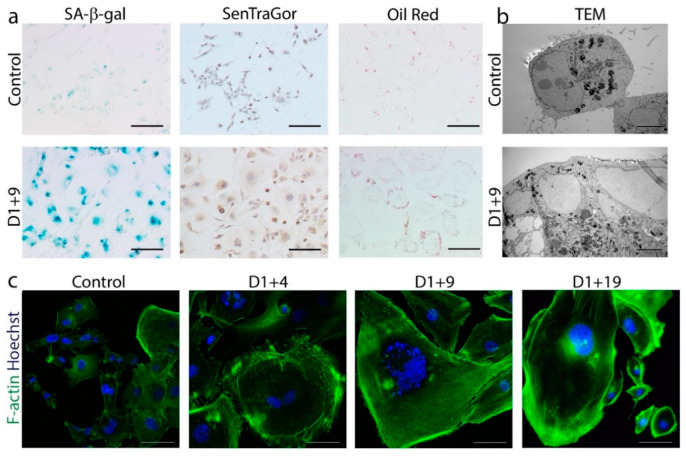
Markers of senescence in dox-treated MDA-MB-231 cells. Cells were treated with 100 nM doxorubicin for 24 h, then cultured in a fresh medium and analyzed on subsequent days. (**a**) Immunocytochemical staining visualized the activity of SA-β-gal (cells stained blue), the accumulation of lipofuscin detected by SenTraGor (cells stained brown) and the accumulation of neutral lipids detected by Oil Red O (red lipid droplets within the cytoplasm). Scale bar: 50 μm. (**b**) Representative transmission electron microscopy images of cross sections showing increased size and number of vacuoles and lipid droplets. Scale bar: 5 µm. (**c**) Representative immunofluorescence images of cell morphology. Cells were stained for F-actin (green), nuclei were stained with Hoechst (blue). Scale bar: 50 μm.

For the induction of cellular senescence in MDA-MB-231 breast cancer cells, we used the same protocol as described previously for colon cancer [30] and in another paper published in the same issue [29] (Figure S1a). Namely, we treated cells for one day (D1) with 100 nM doxorubicin (dox), which yielded the highest number of SA-β-gal-positive cells without a cytotoxic effect. Subsequently, the cells grew in drug-free medium for several days (D1+n). Various cell senescence features were analyzed on day D1, D1+4, D1+9 and D1+19. Figure 1a shows the higher amount of SA-β-gal-positive cells [31], lipofuscin [32] and lipid droplet accumulation [33] in dox-treated cells compared to untreated cells (control). Furthermore, electron (TEM photographs) (Figure 1b) and confocal (F-actin staining) (Figure 1c) microscope images revealed spectacular changes in cell size and an increased number of vacuoles and granules in the cytoplasm of dox-treated cells.

**Figure 2:**
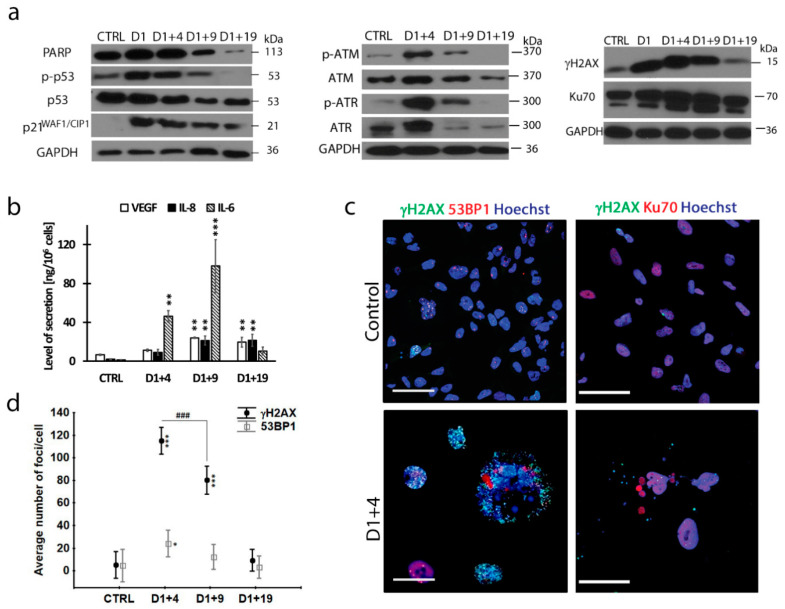
Persistent DNA damage in senescent MDA-MB-231 cells. Cells were treated with 100 nM doxorubicin for 24 h, then cultured in a fresh medium and analyzed on subsequent days. (**a**) Protein level of senescence markers: PARP1 (Poly [ADP-ribose] polymerase 1), p-53, p53 and p21WAF1/CIP1 and elements of DNA damage response: p-ATM, ATM, p-ATR, ATR, γH2AX and Ku70; typical western blot image. (**b**) Amount of IL-8, IL-6 and VEGF (Vascular Endothelial Growth Factor) secreted by cells measured by ELISA. Bars: mean value, error bars: SEM, *n* = 3. (**c**) Representative immunofluorescence images of cells stained for γH2AX (green), 53BP1/Ku70 (red) and nuclei stained with Hoechst (blue). Scale bar: 50 μm. (**d**) Quantification of γH2AX and 53BP1 foci per cell performed using immunofluorescence microscopy. Each point: mean value ± 0.95 confidence interval, *n* = 3. Statistical significance (in relation to control): * *p* < 0.05, ** *p* < 0.01, *** *p* < 0.001, between samples: ### *p* < 0.001.

We also observed transiently higher levels of p-p53 and p21 (Figure 2a, left panel), as well as an increase in SASP components (Figure 2b) in dox-treated cells in comparison with untreated cells, which proves that dox-treated MDA-MB-231 cells undergo cell senescence. A lack of PARP1 (Poly ADP-ribose polymerase 1) cleavage (Figure 2a), the cell viability (Figure S1b) and the morphology showed that the majority of attached cells survived dox treatment. Persistent DNA damage, i.e., DSBs, is considered to be the main culprit of cell senescence [34]. Accordingly, we observed a steep increase in the γH2AX level in dox-treated cells (Figure 2a, right panel). Moreover, the levels of phosphorylated key proteins of DDR, such as ATM (ataxia telangiectasia mutated) and ATR (ataxia telangiectasia and Rad3-related protein), increased substantially on day D1+4 (Figure 2a, middle panel). As these changes were transient and the DDR proteins were practically undetectable on day D1+19, we checked the level of the Ku70 protein and the formation of 53BP1 foci involved in non-homologous end joining (NHEJ) and homologous recombination (HR), respectively [35]. The level of Ku70 (which interestingly also has an important role in human cells to prevent telomere loss [36]), was already high in non-treated cells and increased slightly after dox treatment (Figure 2a, right panel and Figure 2c). Additionally, the average numbers of 53BP1 foci per cell increased after dox treatment but were five times and four times lower than the γH2AX foci number on day D1+4 and D1+9, respectively, suggesting insufficient DNA DSB repair (Figure 2d). Note that, on day D1+19, the number of γH2AX foci decreased dramatically, reaching a value similar to that in untreated cells (about 10 foci/cell), which corresponded with a diminished protein level (Figure 2a,d). Immunostaining revealed that proteins involved in DNA damage repair (53BP1 and Ku70) were preferentially localized in the largest subnuclei of giant cells, in which a relatively small number of γH2AX foci were present (Figure 2c). Conversely, more γH2AX foci were visible in the small nuclear slivers, which seemed to spread from the main nucleus. Altogether, this could suggest that, in giant cells, the DNA repair process is much more efficient in relatively big subnuclei than in small nuclear slivers.

### 2.2. Transient Polyploidization of Doxorubicin-Treated MDA-MB-231 Cells

**Figure 3:**
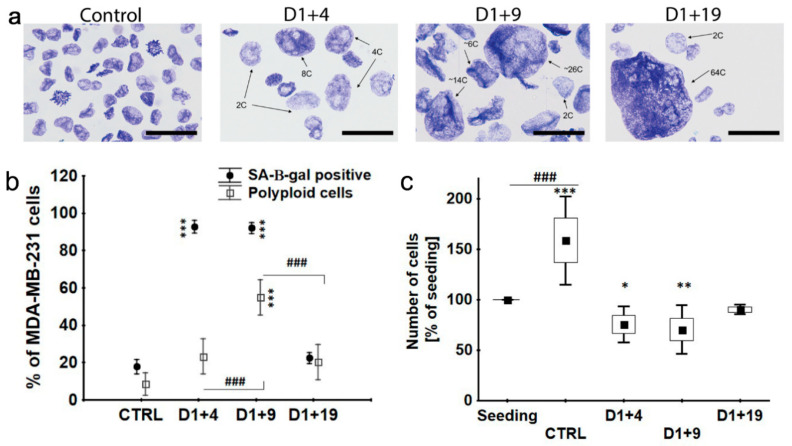
Polyploidy formation and regrowth of senescent MDA-MB-231 cells. Cells were treated with 100 nM doxorubicin for 24 h, then cultured in a fresh medium and analyzed on subsequent days. (**a**) DNA content of cell nuclei estimated by toluidine blue staining. Scale bar: 50 μm. (**b**) Percentage of SA-β-gal-positive cells and polyploid ones. Data are calculated as the percentage of the total cell population. Each point: mean value ± 0.95 confidence interval, *n* = 3. (**c**) Cell number estimated by trypan blue exclusion. Data are calculated as the percentage of the number of seeded cells. Black square: mean, rectangle: mean ± SD, error bars: mean ± 1.96 * SD, *n* = 3. Statistical significance (in relation to control): * *p* < 0.05, ** *p* < 0.01, *** *p* < 0.001, between samples: ### *p* < 0.001.

We analyzed DNA content in dox-treated MDA-MB-231 cells using stoichiometric toluidine blue staining and image cytometry analysis, showing cell polyploidization after dox-treatment [29]. Here, we illustrate the giant cells. As can be seen in Figure 3a on day D1+4, polyploid cells containing ≥4C DNA were present. On day D1+19, some of the nuclei even contained 64C or more DNA. The relative number of polyploid cells containing ≥4C DNA was the highest on day D1+9 when they represented half of the entire cell population (Figure 3b). On day D1+4 and D1+9, about 90% of cells were also SA-β-gal positive (Figure 3b). At the same time, a substantial number of these cells were able to replicate DNA, as proved by a BrdU (Bromodeoxyuridine) incorporation assay (Figure S1c). However, mainly giant nuclei were positive for BrdU (Figure S1d). It suggests that BrdU incorporation is associated with polyploidization of senescent cells rather than the proliferation of a minor population of non-senescent cells. On day D1+19, about 50% of cells were BrdU positive, however, at that time, the number of SA-β-gal-positive cells, similarly to polyploid cells, dropped to 20% of the total population (Figure 3b), while the total cell number increased (Figure 3c). This proves that, on day D1+19, DNA replication was coupled to the cell division of escapers from senescence/polyploidy. Taken together, our data confirmed that dox-induced senescence preceded cell polyploidization; however, the state of senescence/polyploidy was transient and cells regained the ability to divide, along with losing senescence traits. On D1+19, the number of polyploid and SA-β-gal-positive cells resembled those in the control.

### 2.3. Atypical Divisions of Polyploid/Senescent Cells

**Figure 4:**
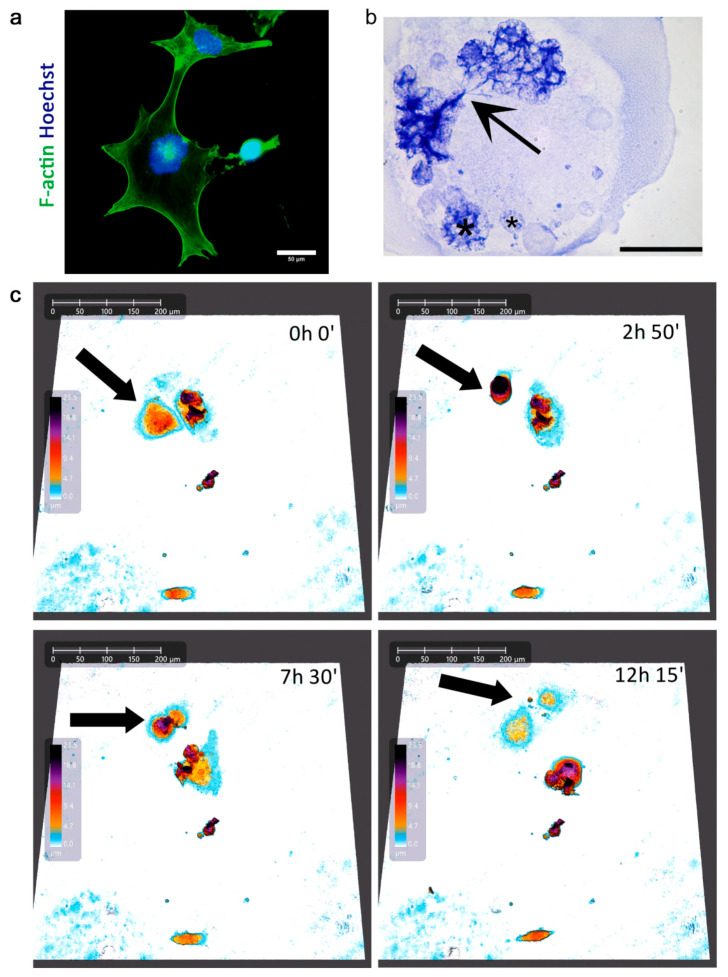
Depolyploidization of a giant senescent MDA-MB-231 cell. Cells were treated with 100 nM doxorubicin for 24 h, then cultured in a fresh medium and analyzed on subsequent days. (**a**) Intermediate state of asymmetric division. Cells were stained for F-actin (green), nuclei were stained with Hoechst (blue). Scale bar: 50 μm. (**b**) Multinucleated polyploidy cell with incomplete mitosis, daughter subnuclei remain linked by a series of chromosome bridges (arrow), smaller interphase nuclei marked with a star (*). Toluidine blue staining at pH 4 after shortened acid hydrolysis. Scale bar: 50 μm. (**c**) Time lapse of asymmetric division of polyploid giant cancer cell on day D1+7, documented with holographic microscope.

In our previous studies, by using an immunostaining method, we showed that giant cells, which originate due to the mitotic slippage, eventually acquired an amoeboid phenotype and bud the depolyploidized progeny, restarting the mitotic cycling [29]. Here, we show some more pictures which illustrate aberrant mitosis and cell budding (Figure 4a,b).

We extended our studies by using live cell imaging, employing two different methods—holographic microscopy (HoloMonitor4, LabSoft, Warsaw, Poland) and scanning disc confocal microscopy (Zeiss, Oberkochen, Germany). Independently of the technique used, we observed the asymmetric divisions of some giant polyploid cells, after which both maternal and descendant cells survived and thrived over the duration of the movies (3–5 days) (Figure 4c, Figure S2b, Videos S1 and S2). For comparison, we performed live cell imaging of control MDA-MB-231 cells, which showed proper, symmetric divisions (Figure S2a). Although these images did not undergo an ameboid-like transition and cell budding, they showed atypical cell divisions of polyploid/giant cells. It seems that different atypical cells divisions, not mutually exclusive, can occur in this cell population, as has been suggested before [37].

### 2.4. Resumption of Autophagic Flux in Escape from Dox-Induced Senescence

**Figure 5:**
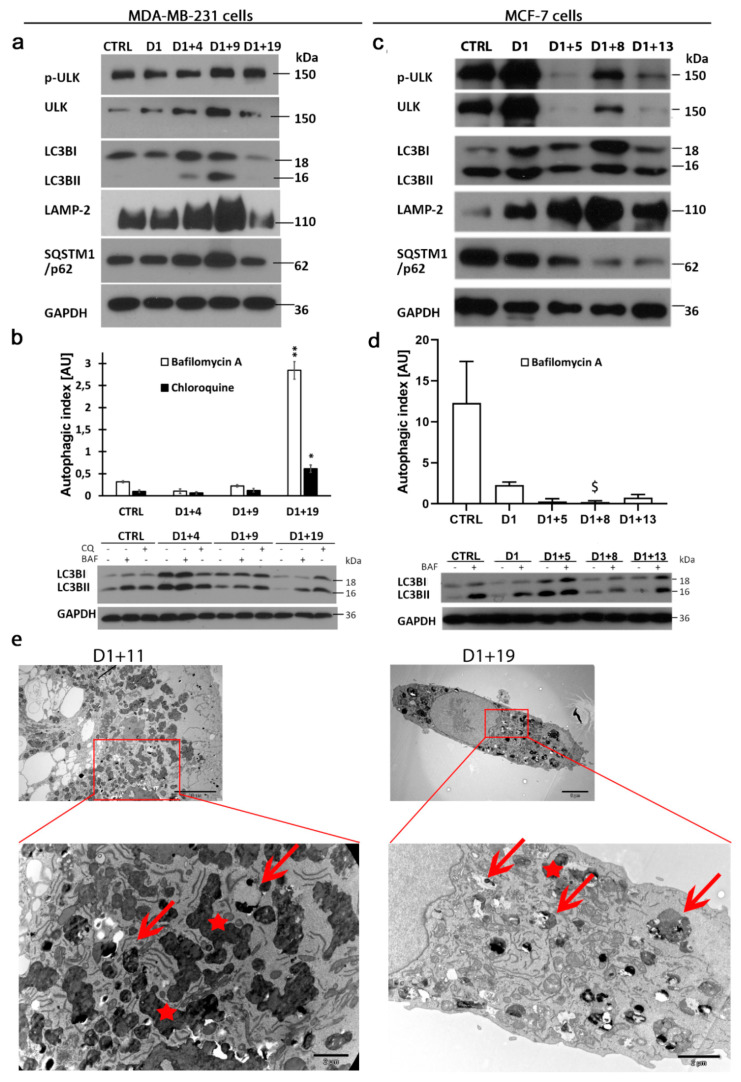
Autophagic flux resumption in descendants of polyploid/senescent MDA-MB-231 and MCF-7 cells. Cells were treated with 100 nM doxorubicin for one day, then cultured in a fresh medium and analyzed on subsequent days. (**a**,**c**) Representative western blots showing autophagy protein levels of p-ULK1 (S757), ULK1, LC3B, LAMP-2 and SQSTM1/p62 in MDA-MB-231 cells (**a**) and MCF-7 cells (**c**). (**b**,**d**) Quantitative analysis of autophagic index based on LC3B protein levels in untreated and bafilomycin A- or chloroquine-treated MDA-MB-231 cells (**b**) and MCF-7 cells (**d**) with representative western blots showing LC3B protein levels. Bars: mean value, error bars: SEM, *n* = 4. Statistical significance (in relation to control): $ *p* < 0.051, * *p* < 0.05, ** *p* < 0.01. (**e**) Transient accumulation of autophagic vesicles. TEM images show typical MDA-MB-231cells on the subsequent days following treatment (upper panel) and their magnified parts with autophagic vesicles (red arrows) and lipofuscin particles (red asterisks).

Descendant cells that appeared after the asymmetric divisions of polyploid/senescent cells were similar to parental cells in size, however, they had increased cellular granularity (Figure S3a,b,d) that might have been a result of different autophagy status. The interconnection of autophagy and senescence is widely reported, although the mechanism of this relation is not fully understood. To elucidate autophagy status in parental cells, dox-induced senescent cells and escapers, several markers of subsequent autophagy phases were analyzed. The mechanistic target of rapamycin complex 1 (mTORC1)- and 5′ adenosine monophosphate-activated protein kinase (AMPK)-dependent signaling pathways are two key regulators of autophagy induction. The inhibition of mTORC1 activity and the increase in AMPK activity are usually correlated with autophagy induction. Nevertheless, autophagy regulation depends on the resulting activity of the effectors of these two signaling pathways, e.g., ULK1. A transient decrease in mTORC1 activity and increased AMPK phosphorylation on day D1+4 (Figure S3c) suggested autophagy induction in senescent/polyploid cells. However, the level of phosphorylated by the mTORC1 form of ULK1 (S757) was unchanged and the levels autophagy effector proteins, such as microtubule-associated protein light chain 3 (LC3B-II), SQSTM1/p62 and LAMP-2, were increased on day D1+4 and D1+9, suggesting autophagy vesicle accumulation (Figure 5a). Such a phenomenon may be a result of either autophagy induction or its inhibition at a late stage. To reveal whether autophagy is induced or inhibited in dox-induced senescent cells, we evaluated the efficiency of new autophagic vesicle formation and degradation by estimation of the autophagic index (AI). It was calculated as the ratio of LC3B-II/LC3B-I protein levels in inhibitor-treated (bafilomycin A or chloroquine) versus untreated cells (Materials and Methods). The autophagic index was very low in the MDA-MB-231 control cells. Interestingly, it was 20 times lower than in colon cancer HCT116 cells and 80 times lower than in normal fibroblasts [28]. The autophagic index remained unchanged or even dropped in MDA-MB-231 senescent cells on day D1+4 and D1+9 (Figure 5b), proving no enhancement of autophagic flux. In light of this, the increased level of LC3B-II in senescent cells may indicate autophagy blockage at the degradation phase. This is in line with SQSTM1/p62 accumulation (Figure 5a) and increased lipid content (Figure 1a). Interestingly, MCF-7 cells with a 25 times higher autophagic index than MDA-MB-231 cells (Figure 5d) had similar vulnerability to dox (Figure S4a) and underwent dox-induced senescence, as we previously described in detail [38]. Additionally, the senescence of MCF-7 cells was coupled with polyploidization, indicated by a simultaneous increase in the number of SA-β-gal-positive and polyploid cells (Figure S4b). Similarly to MDA-MB-231 cells, senescent/polyploid MCF-7 cells were characterized by an increased level of LC3B-II and LAMP-2 (Figure 5c), as well as a decrease in AI (Figure 5d). Hence, senescence is intertwined with autophagy blockage at the degradation phase in both types of cells.

Nevertheless, the appearance of descendants in senescent MDA-MB-231 and MCF-7 cells was accompanied by a decrease in autophagy markers and a significant increase in autophagy activity, measured by autophagic index (Figure 5a–d). Interestingly, in both cell lines, the level of Rubicon protein (a recently recognized endogenous autophagy inhibitor [16]) was transiently increased during the senescence state but returned to the control or even lower level during escape from senescence (on day D1+19/D1+13, Figures S3c and S4d). Moreover, TEM images of MDA-MB-231 cells demonstrated the accumulation of electron-dense, single-membrane autophagic vesicles (red arrows) and lipofuscin particles (red asterisk) on day D1+11, which confirms autophagy blockage at the degradation stage in senescent cells (Figure 5e). Simultaneously, TEM analysis revealed that escapers that appeared on day D1+19 were characterized by a lower number of vesicles with non-degraded cargo or lipofuscin particles.

Together, our results show that autophagic flux in control (parental) MDA-MB-231 cells, although very low, it is sufficient for cell survival and division. Dox-induced senescence is associated with the modulation of autophagy-regulated pathways, such as mTOR and AMPK, however, without visible enhancement in autophagic flux. This may be associated with the impairment of degradation function and abortive autophagy induction in senescent cells. In contrast to this, the appearance of senescence escapers is accompanied by the decrease in autophagic markers and autophagic flux resumption.

### 2.5. Resumption of Autophagic Flux Is Accompanied by Translocation of Transcription Factor EB (TFEB) to Nucleus

**Figure 6:**
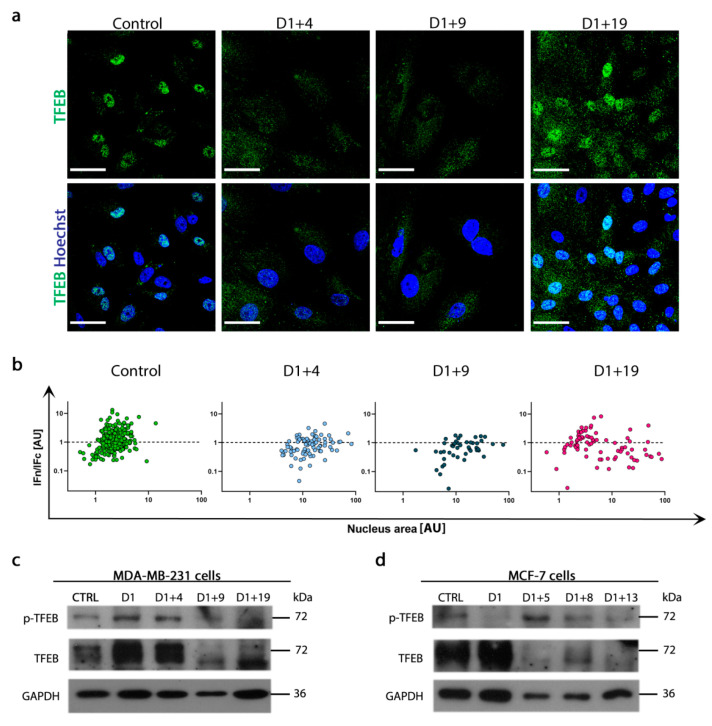
Transcription Factor EB (TFEB) translocation to the nucleus in escapers with functional autophagic flux. (**a**) Representative immunofluorescence images of TFEB (green) protein localization in MDA-MB-231 cells, nuclei stained with Hoechst (blue). Scale bar: 50 μm. (**b**) Quantitative analysis of the intensity of TFEB fluorescence in nucleus/cytoplasm (IFn/IFc) versus nucleus area in MDA-MB-231 cells. (**c**,**d**) Representative western blots showing p-TFEB (S142) and TFEB protein levels in MDA-MB-231 (**c**) and MCF-7 cells (**d**).

The cargo overload of vesicles of lysosomal origin may cause their destabilization and impairment of degradation function. Thus, the increased level of LAMP-2 observed in senescent cells could be the result of either lysosome and autolysosome accumulation or the enhancement of lysosome biogenesis. To reveal this, the intracellular localization of transcription factor EB (TFEB), a crucial regulator of autophagy and lysosomal biogenesis, was analyzed. TFEB translocation into the nucleus results in the upregulation of lysosomal biogenesis, whereas TFEB phosphorylation (Ser142), catalyzed by mTORC1, prevents this translocation and leads to TFEB degradation in the cytoplasm [39,40]. As shown in Figure 6a, TFEB is not present in the nuclei of MDA-MB-231 cells on day D1+4 and D1+9, but appears on day D1+19. The quantitative analysis of TFEB nuclear/cytoplasmic distribution in correlation to nuclear size clearly revealed that senescent cells were characterized by a bigger nucleus area and poorer nuclear localization of TFEB. Moreover, TFEB translocation into the nucleus on day D1+19 was more prominent in cells with small nuclei, similar in size to control cell nuclei, than in big senescent/polyploid cells (Figure 6b). Accordingly, on day D1+19, the autophagic index increased and LC3B-II, LAMP-2 and SQSTM1/p62 protein levels decreased (Figure 5a,b), proving functional autophagic flux in the escapers. As TFEB nuclear/cytoplasmic distribution is regulated mainly by mTORC1, we analyzed the level of TFEB protein phosphorylated by mTORC1. In both cell lines, phospho-TFEB (Ser142) increased in senescent cells and decreased when escapers appeared (Figure 6c,d). This may suggest mTORC1-dependent lysosomal biogenesis and autophagic flux resumption in senescence escapers.

### 2.6. Escapers from Dox-Induced Senescence Were Characterized by Functional Autophagic Flux and Senescence Capability

**Figure 7:**
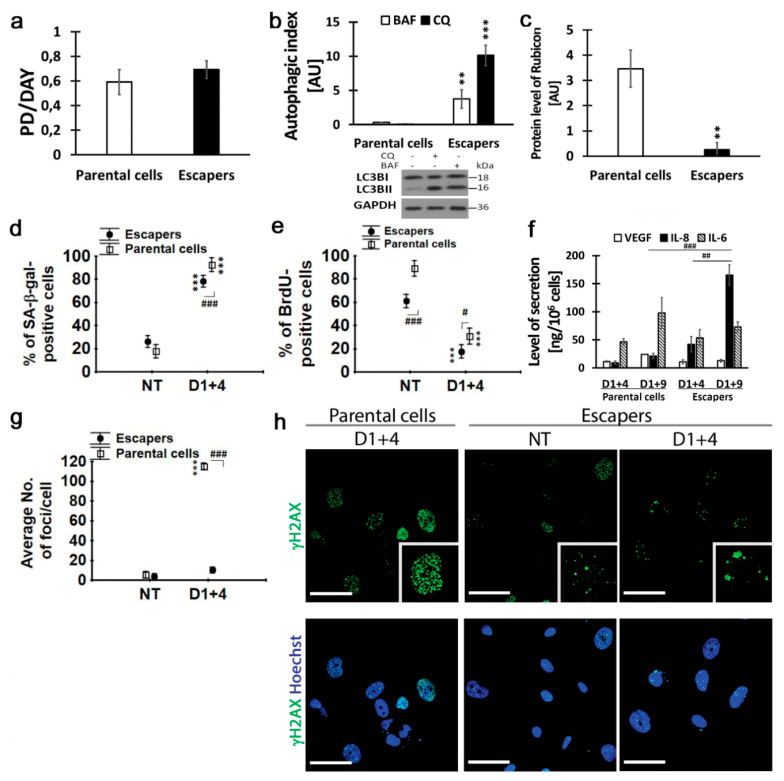
Comparison of parental MDA-MB-231 cells and escapers. Parental cells and escapers were treated with 100 nM doxorubicin for 24 h, then cultured in a fresh medium and analyzed on the 4th day (D1+4). (**a**) Analysis of population doublings per day. (**b**) Quantitative analysis of autophagic index based on densitometry of LC3B protein level in untreated and bafilomycin A (BAF)- or chloroquine (CQ)-treated cells. (**c**) The protein level of Rubicon; densitometry analysis of western blot bands from four independent experiments. (**d**,**e**) Percentage of SA-β-gal- and BrdU-positive cells. Data are calculated as the percentage of the total cell population. (**f**) Amount of IL-8, IL-6 and VEGF secreted by cells on consecutive days after doxorubicin treatment. (**g**) The average number of γH2AX foci per cell. (**h**) Representative immunofluorescence images of parental cells and escapers stained for γH2AX (green) and Hoechst (blue). Scale bars: 50 μm. Statistics: bars: mean value, error bars: SEM, each point: mean value ± 0.95 confidence interval, *n* = 4. Statistical significance (in relation to non-treated (NT) or parental cells): ** *p* < 0.01, *** *p* < 0.001, between samples: # *p* < 0.05, ## *p* < 0.01, ### *p* < 0.001.

We cultured escapers for one month beyond day D1+19 or D1+13 (for MDA-MB-231 or MCF-7 cells, respectively) and characterized them in terms of DNA content, senescence susceptibility and autophagic flux. As we mentioned before, MDA-MB-231 cells which escaped from senescence were slightly enlarged and their granularity was increased in comparison to parental cells (Figure S3a,b,d). However, analysis of the DNA content revealed no significant differences between parental cells and escapers (DNA index equaled 1.616 and 1.614, respectively), indicating the near-triploid karyotype characteristic for this cell line. Additionally, the population doublings per day were similar (Figure 7a). Interestingly, these escapers were characterized by high autophagic flux, which was 13 times higher than in parental cells (Figure 7b), and by a 14 times lower protein level of the newly recognized autophagy inhibitor, Rubicon [16] (Figure 7c). Additionally, the amounts of autophagic vesicles with lipofuscin and non-degraded cargo were decreased in escapers (Figure S3d).

Considering that the comparison of MDA-MB-231 parental cells and escapers revealed significant differences in the autophagic index, we were interested in whether enhanced autophagy in escapers may influence their ability to senesce. We used the same experimental schedule to induce senescence by dox in escapers and analyzed them on day D1+4 and D1+9. We found that up to 78% of escapers (slightly less than parental cells) were SA-β-gal positive and less than 20% of cells incorporated BrdU (Figure 7d,e). They underwent polyploidization similarly to parental cells (not shown). The secretion of a chemokine, IL-8, and cytokine, IL-6, was also increased in dox-treated escapers (Figure 7f), however, the pattern of cytokine secretion was different in dox-treated parental cells. Surprisingly, escapers were characterized by only a slight increase in the number of γH2AX foci per cell (from four to 10 foci) after dox treatment, in contrast to one hundred and twenty foci observed in senescent parental cells. Additionally, γH2AX foci induced by dox in escapers were larger and more clustered than in parental cells (Figure 7g,h). It may have been due to properly functioning autophagy and, consequently, more efficient damage removal. Together, escapers were more sensitive to dox and the percentage of escapers which underwent senescence was slightly smaller than for parental cells. However, escapers had increased autophagic flux and they secreted more proinflammatory cytokines. Taken together, ours data and the recent data of others demonstrate that senescent polyploid cells may represent a newly recognized mechanism for the birth of new neoplastic life [41].

In the case of MCF-7 cells, the escapers were characterized by significantly higher population doubling than in parental cells (Figure S4b). The autophagic index and the Rubicon protein level did not differ between these cells, however, basal autophagy in MCF-7 cells, either in parental cells or escapers, was relatively high (Figure S4c,d). Nevertheless, MCF-7 escapers also underwent senescence after pulse dox treatment and an increased number of SA-β-gal-positive cells and decreased BrdU incorporation confirmed the presence of senescence markers on day D1+5 (Figure S4e,f).

Together, our result showed that escapers are different from parental cells not only in terms of autophagy but also size, cellular morphology and secretion. However, we showed that both MDA-MB-231 and MCF-7 escapers were susceptible to dox-induced cell senescence, uncoupled from the autophagy status of these cells.

## 3. Discussion

The induction of polyploid giant cells by anticancer therapy and their role in cancer resistance and metastasis has been well described, however, the issue of polyploid cell senescence has still not been extensively explored by researchers [8,42,43,44]. The depolyploidization of polyploid/multinucleated giant cells was first reported almost twenty years ago [45,46]. These and subsequent studies by Erenpreisa and colleagues demonstrated that treated cells first undergo polyploidization, ultimately resulting in the emergence of mitotically dividing para-diploid progeny [5,47]. Similarly to therapy-induced polyploidization (TIP), it is now generally accepted that therapy-induced senescence (TIS) favors escaping from division arrest and re-emergence into an actively reproductive state. Indeed, many anticancer treatments induce cell senescence both in vitro and in vivo, posing a potential threat to the effectiveness of therapy (reviewed in [48]). Although studies which show the coupling of TIP and TIS are still scarce (reviewed in [44])—it seems logical to assume that these two processes occur simultaneously or sequentially in cancer cells subjected to anticancer therapy and that DNA over-replication in senescent cells is a driving force leading to atypical cell divisions. Indeed, in this study and in a previous one [38], we showed that MDA-MB-231 and MCF-7 breast cancer cells displayed the features of cell senescence, such as SA-β-gal activity and became polyploid. Additionally, the hallmarks of cell senescence, including DNA damage and DNA damage response, were detected several days after dox treatment and they preceded cell polyploidization. Eventually, polyploid cells disappeared from the culture and, in their place, small cells appeared.

DNA damage, especially DSBs and the subsequent DDR, are almost universal features of radio- and chemotherapy treatment [49] and cell senescence [18,38]. The key player in the DDR and senescence is the p53 protein, which transactivates the CDKN1A gene, producing the main cell cycle inhibitor, p21WAF1/CIP1 [6]. Therefore, we used two different breast cancer cell lines, namely MDA-MB-231 and MCF-7, with different p53 statuses. MCF-7 breast cancer cells express WT p53, whereas MDA-MB-231 cells possess a mutated form of TP53 (R280K). In MDA-MB-231 cells, we observed a high level of p21WAF1/CIP1 until day D1+19, when the majority of cells were again proliferating; the escapers, however, reverted to the state of non-detectable p21WAF1/CIP1. The R280K mutation affects the DNA-binding domain [50] and results in the decreased activation and repression of p53 target genes, including CDKN1A [51]. Although we observed p53 phosphorylation, as well as phosphorylation of its upstream activator, ATM, the signal could not be transduced downstream from p53. Thus, the upregulation of p21WAF1/CIP1 observed by us was p53 independent, which is what we previously observed in dox-treated p53-deficient colon cancer cells [52].

Dox-treated MDA-MB-231 cells had a very high number of γH2AX foci, sensing DSBs, and a relatively low number of 53BP1 foci. Both proteins, involved in the DDR, are markers of senescence and take part in homologous recombination (HR), an error-free process dependent on properly functioning autophagy [53,54]. A higher number of 53BP1 than γH2AX foci was considered as a marker of the initiation of DNA repair [55]. However, 53BP1 is also involved in alternative lengthening of telomeres (ALT) [56], which has been shown to participate in the senescence/polyploidization of dox-treated MDA-MB-231 cells [29]. Thus, insufficient autophagy may be the cause of the inability of the MDA-MB-231 cells to repair DNA lesions. However, the Ku70- DNA-dependent protein kinase (DNA PKs) axis, belonging to the non-homologous end joining (NHEJ) pathway, performs DNA repair independently from autophagy [53,57]. In MDA-MB-231 cells, the protein level of Ku70 was slightly upregulated on days D1+4 and D1+9 (Figure 2a), suggesting an active DNA repair process and possibly telomere stabilizing ALT [29]. However, NHEJ is an error-prone mechanism of DNA repair. It was documented that damaged DNA is sorted to the cytoplasm, and probably digested in the process of active autophagy [29]. The very low autophagic index we observed suggested to us abortive autophagy, but does not exclude, however, the active selective autophagy of damaged DNA in senescent/polyploid cells. Nonetheless, this issue needs more studies.

The crucial question concerns the molecular and cellular mechanisms of polyploidization/depolyploidization of breast cancer cells and the phenotype of the escapers. It seems that the paper already published in a Special Issue finally confirmed the role of mitotic slippage in reversible polyploidization in cancer cells induced to senescence. Moreover, the results of that study refreshed the old idea about the role of recapitulation in the amoeba-like agamic lifecycle, decreasing the mutagenic load and enabling the recovery of recombined, reduced progeny for a return to the mitotic cycle [29]. Using a time-lapse, we were able to confirm atypical divisions of giant MDA-MB-231 cells. Furthermore, we also proved that the escapers were not cells that simply avoided senescence/polyploidy and restarted cell division after the drug withdrawal. Live cell images and movies support our claim that escapers are derived from giant polyploid/senescent cells.

The interesting question emerges whether reversible senescence leads to the production of progeny with a different phenotype than parental cells. To date, published data have mainly focused on the differences in stemness and aggressiveness between mother cells and their progeny [4,5,13,58,59,60].

The main question we posed in these studies concerned the role of autophagy in breast cancer cells undergoing reversible senescence/polyploidization. In particular, there is a plethora of evidence showing that the majority of conventional therapies used to combat breast cancer induce autophagy [61]. As mentioned previously, Erenpreisa’s group suggested autophagy as a crucial factor in depolyploidization [20] and the emergence of vital daughter cells via mitotic slippage [21]. Recently, Jakhar et al. documented that postmitotic slippage, leading to tetraploidy formation in cancer cells, depended on autophagy induction. Furthermore, the pharmacologic inhibition of autophagy or the silencing of an autophagy-related gene, ATG5, led to a bypass of G1 arrest senescence, reduced SASP-associated paracrine tumorigenic effects and increased DNA damage after S-phase entry with a concomitant increase in apoptosis [62]. Similarly, Was et al. showed that the pharmacological inhibition of autophagy (by bafilomycin A1) in cancer cells induced to senescence increased cell death, but they claimed that polyploid/senescent cells were resistant to bafilomycin A1 treatment [63]. Moreover, it seems that escaping from senescence needs autophagy reactivation. We showed that senescent/polyploid cells with a very low autophagic index gave rise to either progeny with autophagic indexes that were relatively high (in MDA-MB-231 cells) or similar to the control (in MCF-7 cells). We suggest that the senescence of breast cancer cells is intertwined with insufficient autophagy, whereas autophagy activation is indispensable for the appearance of vital progeny.

Indeed, we have found that general autophagy, estimated by the autophagic index, was impaired during the senescence/polyploidy of breast cancer cells. On the other hand, the transient increase of phosphorylated AMPK and the decrease in phosphorylated mTOR observed in MDA-MB-231 cells suggested autophagy induction, but it was not accompanied by a decrease in mTOR-dependent ULK1/2 phosphorylation (involved in autophagy induction [64]). Altogether, this could suggest that, in senescent/polyploid cells, contradictory processes can be involved in autophagy regulation. Namely, the activation of signals for autophagy induction and the impairment of autophagic flux, leading to a sort of abortive, poorly functioning autophagy process. However, this does not necessarily exclude the activity of selective autophagy, especially in the process of the elimination of damaged DNA, which was confirmed by a low, but still present, autophagic flux. Additionally, in the case of insufficient autophagy degradation, autophagic vesicles with cargo can be removed by extrusion. Moreover, the autophagic index was measured in the entire population of senescent/polyploid cells and the presence of a limited population of cells with high autophagic flux, which produced the progeny with fully functional autophagy, cannot be excluded. Nevertheless, we can conclude that the appearance of escapers in the population of senescent cancer cells is associated with mTOR phosphorylation, however, without the transduction of signal to its substrate, which may be a cause of TFEB translocation to the nucleus. Moreover, the level of the endogenous autophagy inhibitor, Rubicon, also showed transient changes in dox-treated breast cancer cells. The significant increase in Rubicon in senescent cells and then its decrease to or below the level observed in control cells clearly revealed autophagic flux blockage followed by its resumption during escape from senescence.

The relationship between autophagy and cell senescence seems to be complicated, especially in senescent cancer cells as, generally, cancer cells have different basal levels of autophagy, including high and insufficient autophagy [65]. Indeed, MDA-MB-231 cells have a very low autophagic index in comparison to MCF-7 cells and normal fibroblasts [28]. Interestingly, the inhibition of autophagy can promote [66], delay [67] or alleviate senescence [68]. However, our results demonstrated that, regardless of basal autophagic flux, during dox-induced senescence/polyploidization, autophagy was impaired and then increased in escapers. The autophagic index was elevated to the level observed in parental cells in MCF-7 cells or significantly higher in the case of MDA-MB-231 cells.

Moreover, we showed that MDA-MB-231 and MCF-7 parental cells, as well as escapers, underwent drug-induced senescence, although with different efficiencies. That allowed us to conclude that autophagy is not indispensable for senescence induction in MDA-MB-231 cells (with mutated p53 and a very low autophagic flux) and MCF-7 cells (with wild-type p53 and a high autophagic flux). Importantly, our results allowed us to conclude that unlocked autophagy was necessary to release descendants. Furthermore, we proved that the escapers were different from parental cells in terms of autophagy functionality, which is an obvious novelty of our studies. It is worth mentioning that the first study that pinpointed the involvement of autophagy in depolyploidization was published by Erenpreisa’s group [69]. The authors hypothesized that autophagy was responsible for the degradation of chromosome bridges and the release of daughter cells, due to the distribution of cathepsin B in the central cytoplasmic area between subnuclei.

In short, our studies suggest that breast cancer cells can undergo drug-induced senescence, independently from the autophagy status. Furthermore, senescence is intertwined with insufficient autophagy. In turn, transient senescence ensured favorable conditions for the appearance of polyploid cells. However, the appearance of vital progeny is interconnected with functional autophagy. The mechanism of that phenomenon still needs to be unraveled. Although escapers had a similar DNA index to parental cells, they were characterized by a different phenotype. We are the first group to report that reversible polyploidization, intertwined with senescence escape, stably activates autophagic flux due to TFEB translocation to the nucleus and the reduction of the autophagy inhibitor, Rubicon.

## 4. Materials and Methods

Primary antibodies are listed in Table 1. Secondary antibodies: anti-rabbit Alexa 488, anti-mouse Alexa 488, anti-rabbit Alexa 555, anti-mouse Alexa 555 from Life Technologies, (Carlsband, CA, USA), (1:500; A11008, A110296, A21428 and A21422) and anti-guinea pig Alexa 594 from Jackson ImmunoResearch (Cambridgeshire, UK), (1:500; 103-605-155).

### 4.1. Cell Culture and Treatment

MDA-MB-231 (HTB-26) cells, obtained from the European Collection of Authentic Cell Cultures (ECACC, Wiltshire, UK), were kindly provided by Prof. Jekaterina Erenpreisa (Latvian Biomedical and Research Centre, Riga, Latvia). MCF-7 cells (HTB-22) were purchased from the American Type Culture Collection (ATCC). Cells were grown under standard conditions (37 °C, 5% CO_2_) in DMEM low-glucose (MCF-7; Sigma-Aldrich, St. Louis, MO, USA, D5546) or DMEM high-glucose (MDA-MB-231; Biowest, Nuaillé, France, L0104) medium supplemented with 10% fetal bovine serum (FBS) (Cytogen, Zgierz, Poland, S181H), antibiotic–antimycotic solution (Sigma-Aldrich, St. Louis, MO, USA, A5955) and, in the case of, DMEM low-glucose medium, 2 mM l-Glutamine solution (Sigma-Aldrich, St. Louis, MO, USA, G7513) was added. The cells were seeded 24 h before treatment at a density of 1 × 10^4^ cells/cm^2^. To induce senescence, cells were treated with 100 nM dox (IC30; Sigma-Aldrich, St. Louis, MO, USA, D1515) for 24 h and then cultured in fresh medium without the drug for several days (1 + n). Every third day, the medium was replaced by a fresh one. The escaper cell line was established after a monthly culture of small cells collected on D1+19 for MDA-MB-231 cells and on D1+13 for MCF-7 cells in four independent experiments.

### 4.2. Western Blotting Analysis

Alive, adherent cells were harvested and subjected to the procedure described previously [11,63].

### 4.3. Immunocytochemistry

Staining was performed as described in [70,71]. According to requirements, F-actin was stained by additional incubation with phalloidin (1:50; Thermo Fisher Scientific, Waltham, MA, USA, A12379) for 30 min.

### 4.4. Detection of Senescence-Associated β-galactosidase

Staining was performed according to Dimri et al. [31].

### 4.5. Lipofuscin Staining

To detect lipofuscin accumulation, SenTraGor (Arriani Pharmaceuticals, Athens, Greece, AR8850020) staining was performed as described by Evangelou et al. [72].

### 4.6. Lipid Staining

Cells grown on coverslips were fixed in 4% paraformaldehyde (PFA) (Sigma-Aldrich, St. Louis, MO, USA, P6148) for 15 min at room temperature. Then, cells were washed with ddH2O, dehydrated (5 min incubation in 60% isopropanol) and stained with 0.3% Oil Red O (Sigma-Aldrich, St. Louis, MO, USA, O-0625) in 60% isopropanol for 7 min. The Oil Red O solution was discarded, stained cells were washed with ddH2O and covered with mounting medium.

### 4.7. Cytokine Measurement

To assess the secretion of IL-8, IL-6 and VEGF proteins, the culture medium was collected and subjected to analysis by the DuoSet ELISA Development Kit, according to the manufacturer’s instructions (R&D Systems, Minneapolis, MN, USA, DY208-05, DY206-0, DY293B-05). Absorbance was measured using a Tecan Sunrise microplate reader (Tecan Group Ltd., Männedorf, Switzerland).

### 4.8. DNA Content Evaluation by Toluidine Blue Staining

For DNA content analysis, stoichiometric DNA staining with toluidine blue (TB; Fisher Scientific, Waltham, MA, USA, T161-25) was performed as described previously [73]. As a reference for locating normal 2C peaks, the nuclei of leukocytes were used. The estimated integral error of the method was lower than 10%.

### 4.9. Bromodeoxyuridine Incorporation Assay

DNA synthesis assay was performed as described previously [52].

### 4.10. Cell Size and DNA Index Estimation by Flow Cytometry Analysis

Cell size and cell granularity were determined with the use of flow cytometry as described previously [52]. DNA index was determined with the use of peripheral blood mononuclear cells as a diploid reference.

### 4.11. TEM Sample Preparation

Cells growing on a 35-mm glass bottom dish (MatTek, P35G-1.5-14-CGRD) were fixed with 2% paraformaldehyde (Sigma Aldrich, P6148) and 1% glutaraldehyde (EMS, EM grade) in 0.2 M HEPES pH 7.3 and prepared for electron microscopy according to a published protocol, with minor changes [74]. Briefly, cells were post-fixed with 1% aqueous solution of osmium tetroxide (Agar Scientific, AGR1023) and 1.5% potassium ferrocyanide (Sigma Aldrich, St. Louis, MO, USA, P3289) in PB for 30 min on ice. Then, samples were immersed in 1% aqueous thiocarbohydrazide (Sigma Aldrich, St. Louis, MO, USA, #88535) for 20 min, post-fixed with 2% aqueous solution of osmium tetroxide for 20 min (all at room temperature) and incubated in 1% aqueous uranyl acetate at 4 °C overnight. The next day, samples were exposed to 0.66% lead aspartate for 30 min at 60 °C, dehydrated with increasing dilutions of ethanol, infiltrated with Durcupan resin (Sigma Aldrich, St. Louis, MO, USA, #44610), embedded using a BEEM capsule according to a published protocol [75] and hardened at 70 °C for at least 72 h. The resin blocks were cut with an ultramicrotome (ultracut R, Leica) and ultrathin sections (70 nm) were collected on formvar-coated copper grids, mesh 100 (Agar Scientific, AGS138-1).

### 4.12. Population Doubling (PD)

To determine the cell number parameters, cells were counted with a Neubauer camera by trypan blue dye exclusion. The quantification of PD as a measure of cell growth for each cell line was carried out on the basis of the total number of viable cells.

### 4.13. Double Staining

The double staining method with Hoechst 33342/PI [76] was used in the investigation of the effect of doxorubicin on the MDA-MB-231 cells.

### 4.14. Autophagic Index

To quantify autophagic flux in the subsequent days following treatment, we calculated the autophagic index (AI). This was achieved by establishing the difference between the ratio of the LC3B II/LC3B I protein level (ΔLC3B) of cells treated with 200 nM bafilomycin A (Sigma-Aldrich, St. Louis, MO, USA, B1793) or 50 μM chloroquine (Lab Empire, Rzeszów, Poland, CHL919) for the last 3 h of culture and untreated ones and normalizing it to the ΔLC3B of untreated ones according to the following equations: for bafilomycin A, AIBAF = (ΔLC3BBAF − ΔLC3BNT)/ΔLC3BNT and for chloroquine, AICQ = (ΔLC3BCQ − ΔLC3BNT)/ΔLC3BNT.

### 4.15. Live Imaging

To pinpoint the origin of escaper cells, live imaging techniques for the division of giant polyploidy MDA-MB-231 cells were employed. Two independent techniques were used: a holographic microscope, HoloMonitor4 (LabSoft, Warsaw, Poland) and a spinning disc confocal microscope Zeiss Axio Observer Z.1 Inverted Microscope (Zeiss, Oberkochen, Germany) with Yokogawa CSU-X1 Spinning Disc (Yokogawa, Tokyo, Japan), with objective: C APO 40x/1.20 Water. Film acquisition took 3–5 days, time between each frame: 15–30 min.

### 4.16. Image Acquisition

Immunofluorescence (IF) specimens were visualized either with a Nikon Eclipse Ti (Tokio, Japan), a fluorescent microscope with a 40×/0.6 Nikon lens, or a confocal laser scanning microscope, Leica TCS SP8 (Wetzlar, Germany), usingHC PL APO CS2 63x/1.40 Oil immersion lens. Electron microscope (EM) specimens were imaged with a transmission electron microscope, JEM 1400 (JEOL Co., Tokyo, Japan, 2008), equipped with a 11 megapixel TEM camera MORADA G2 (EMSIS GmbH, Münster, Germany). Digital images of nuclei stained with toluidine blue were collected using a Sony DXC 390P color video camera.

### 4.17. Quantitative Analysis

Computational analyses of cell, foci number and fluorescence intensity were performed using ImageJ (FiJi) software. The ratio of the intensity of TFEB fluorescence in the nucleus versus the cytoplasm was measured as corrected total cell fluorescence (CTCF), according to the equation: CTCF = integrated density of selected area (nucleus/cytoplasm) − (selected area (nucleus/cytoplasm)) × mean fluorescence of background readings).

DNA content was measured as the integral optical density (IOD), using Image-Pro Plus 4.1 software (Media Cybernetics, Rockville, MD, USA). More than 50 cells were counted per sample in each analysis.

### 4.18. Statistical Analysis

Sample size was chosen according to previous observations, in which similar experiments were performed in order to see significant results, in this case with heterogeneous biological replicates. Therefore, all presented data concerning parental and escapers cells are shown as an average of at least three or four independent experiments. All biological replicates without exclusion were used to perform statistical analysis. In the case of bar graphs, the error bars represent the SEM, whereas in ANOVA graphs, the vertical bars indicate a 0.95 confidence interval. Statistical analysis was performed with the use of the STATISTICA 11 program (TIBCO Software Inc., Palo Alto, CA, USA) or GraphPad Prism 8 (San Diego, CA, USA). ANOVA (analysis of variance), analysis was used for the analysis of differences among three or more groups, followed by post hoc analysis (Tukey’s honest significant difference test; HSD test). Multiple comparisons were done after a homogeneity test for variance. Variance was similar between the groups that were being statistically compared. Normal distribution of the data was tested with a Shapiro–Wilk test. Statistical significance in relation to the control is marked with an asterisk (* or $), whereas that between subsequent days of treatment is shown with a hash (#). The *p*-value is stated as: $ *p* < 0.051, * 0.01 < *p* < 0.05, ** 0.001 < *p* < 0.01, *** *p* < 0.001.

## Figures and Tables

**Table 1:** Primary antibodies used in the study.

	Description	Concentration	Product No. and Manufacturer
PARP1	Mouse monoclonal	1:500	#556494, BD Bioscences, Franklin Lakes, NJ, USA
p53 (DO-1)	Mouse monoclonal	1:500	sc-126, Santa Cruz, Dallas, TX, USA
p-p53 (S15)	Rabbit polyclonal	1:500	#9284, Cell Signaling Technology, Danvers, MA, USA
p21 WAF1/CIP1	Mouse monoclonal	1:500	P1484, Sigma-Aldrich, St. Louis, MO, USA
ATM (Y170)	Rabbit monoclonal	1:500	ab32420, Abcam, Cambridge, UK
p-ATM (S1981)	Mouse monoclonal	1:500	ab36810, Abcam, Cambridge, UK
ATR	Rabbit polyclonal	1:500	#2790, Cell Signaling Technology, Danvers, MA, USA
p-ATR (S428)	Rabbit polyclonal	1:500	#2853, Cell Signaling Technology, Danvers, MA, USA
Ku70 (E-5)	Mouse monoclonal	1:500	sc-17789, Santa Cruz, Dallas, TX, USA
ὙH2AX (S139)	Mouse monoclonal	1:1000	ab26350, Abcam, Cambridge, UK
ὙH2AX (S139)	Rabbit monoclonal	1:500	#9718, Cell Signaling Technology, Danvers, MA, USA
53BP1	Rabbit polyclonal	1:500	NB100, Novus Biologicals, Centennial, CO, USA
Ki67	Rabbit polyclonal	1:500	ab15580, Abcam, Cambridge, UK
SQSTM1/p62	Mouse monoclonal	1:1000	#610832, BD Bioscences, Franklin Lakes, NJ, USA
SQSTM1/p62	Guinea pig polyclonal	1:500	GP62-C, Progen, Heidelberg, Germany
LC3B	Rabbit polyclonal	1:500	L7543, Sigma-Aldrich, St. Louis, MO, USA
ULK1 (D8H5)	Rabbit monoclonal	1:250	#8054, Cell Signaling Technology, Danvers, MA, USA
p-ULK1 (S757)	Rabbit monoclonal	1:250	#14202, Cell Signaling Technology, Danvers, MA, USA
m-TOR (7C10)	Rabbit monoclonal	1:500	#2983, Cell Signaling Technology, Danvers, MA, USA
p-m-TOR (S2448)	Rabbit monoclonal	1:500	#5536, Cell Signaling Technology, Danvers, MA, USA
p70S6K	Rabbit monoclonal	1:500	#2708, Cell Signaling Technology, Danvers, MA, USA
p-p70S6K (T389)	Rabbit monoclonal	1:500	#9234, Cell Signaling Technology, Danvers, MA, USA
p-S6 (S235/236)	Rabbit monoclonal	1:500	#4858, Cell Signaling Technology, Danvers, MA, USA
4EBP1	Rabbit polyclonal	1:500	#9452, Cell Signaling Technology, Danvers, MA, USA
p-4EBP1 (T37/46)	Rabbit monoclonal	1:500	#2855, Cell Signaling Technology, Danvers, MA, USA
p-AMPK(T172)	Rabbit monoclonal	1:500	#2531, Cell Signaling Technology, Danvers, MA, USA
AMPK	Rabbit monoclonal	1:250	#2532, Cell Signaling Technology, Danvers, MA, USA
p-Akt(S473)	Rabbit monoclonal	1:2000	#4060, Cell Signaling Technology, Danvers, MA, USA
Akt	Rabbit monoclonal	1:1000	#4691, Cell Signaling Technology, Danvers, MA, USA
LAMP-2	Mouse monoclonal	1:500	#14-1078-82, eBioscience, Thermo Fisher Scientific, Waltham, MA, USA
TFEB	Rabbit polyclonal	1:250	#4240, Cell Signaling Technology, Danvers, MA, USA
p-TFEB (S142)	Rabbitpolyclonal	1:500	ABE-1971, Merck Millipore, Burlington, MA, USA
Rubicon	Rabbit monoclonal	1:500	#8465, Cell Signaling Technology, Danvers, MA, USA
GAPDH	Mouse monoclonal	1:50,000	MAB374, Merck Millipore, Burlington, MA, USA

## Supplementary Materials

Supplementary materials can be found at https://www.mdpi.com/1422-0067/21/17/6084/s1. Figure S1: Doxorubicin effect on MDA-MB-231cells, Figure S2: Analysis of division of control MDA-MB-231 cells and cells treated with dox at D1+12, Figure S3: Analysis of MDA-MB-231 cells escaping senescence, Figure S4: Analysis of MCF-7 parental cells and cells escaping senescence, Video S1: Time lapse of asymmetric division of polyploid giant MDA-MB-231 cell on day D1+7, documented with holographic microscope, Video S2: Time lapse of asymmetric division of polyploid giant MDA-MB-231 cell on day D1+12, documented with confocal spinning disc microscopy.

## Abbreviations

AI	Autophagy Index
DDR	DNA Damage Response
DOX	Doxorubicin
DSBs	Double-Strand Breaks
HR	Homologous Recombination
NHEJ	Non-Homologous End Joining
SA-β-gal	Senescence-Associated β-galactosidase
SASP	Senescence-Associated Secretory Phenotype
TIP	Therapy-Induced Polyploidy
TIS	Therapy -Induced Senescence
