# Supplementary material for: Improved Autophagic Flux in Escapers from Doxorubicin-Induced Senescence/Polyploidy of Breast Cancer Cells
Source: Int J Mol Sci. 2020 Aug 24;21(17):6084. doi: 10.3390/ijms21176084 (PMC7504443; doi:10.3390/ijms21176084)
Supplement: Supplementary file 1 [file ijms-21-06084-s001.zip › Supplementary Figures_R2.pdf]

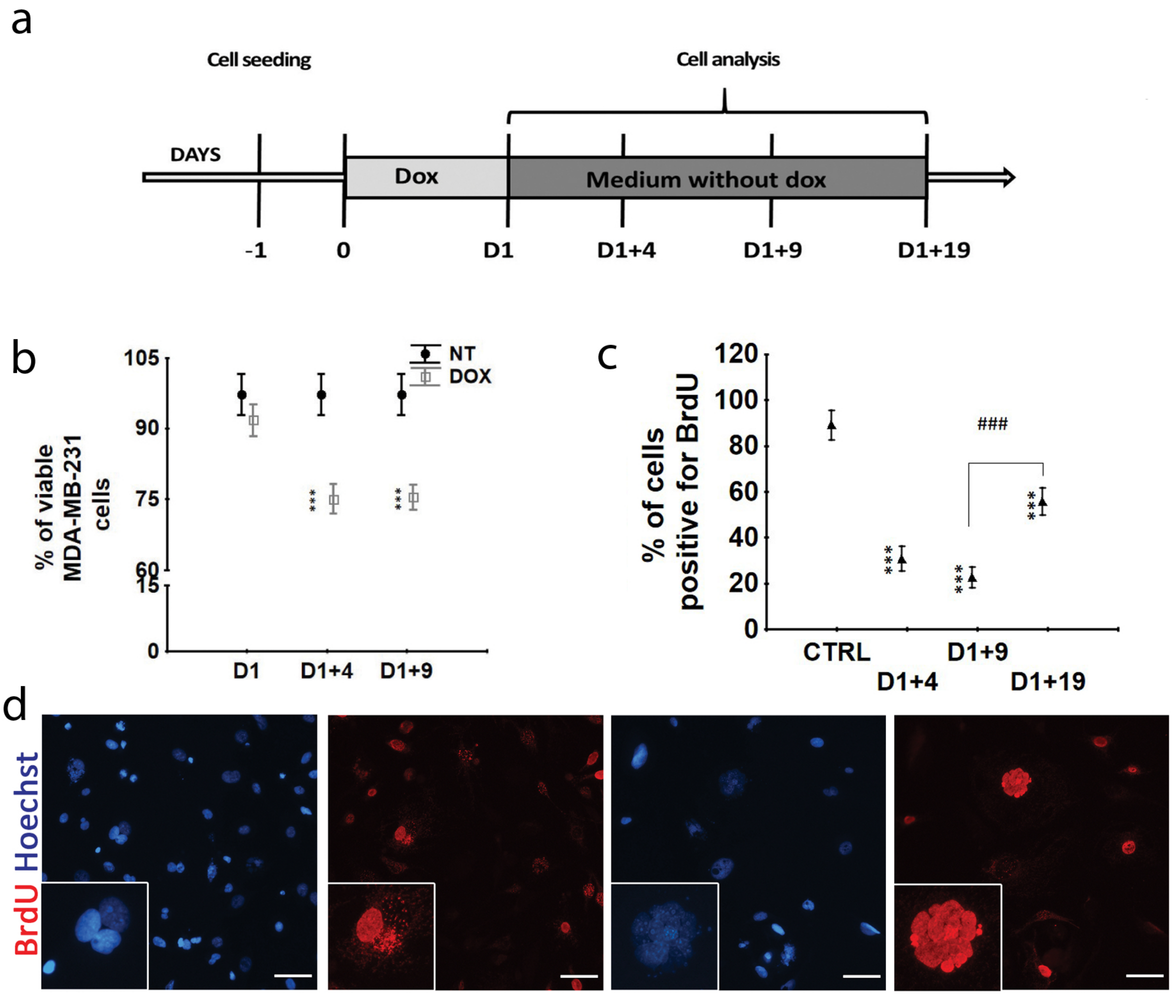

Figure S1. Doxorubicin effect on MDA-MB-231 cells. a:Scheme of doxorubicin treatment. b:Viability of cells on consecutive days after doxorubicin treatment. Data obtained by PI and Hoechst 33342 double staining. Each point: mean value+0.95 confidence interval, N=3. c: Percentage of cells positive for BrdU. Data are calculated as the percentage of the total cell population. d: Representative immunofluorescence images of giant nuclei (blue) stained with BrdU (red).Not all nuclei in multinucleated cell are BrdU positive. Scale bars: 20 um. b-c: Statistical significance (in relation to D1 or control): p<0.05-*, p<0.01-**, p<0.001-***, #-between samples.

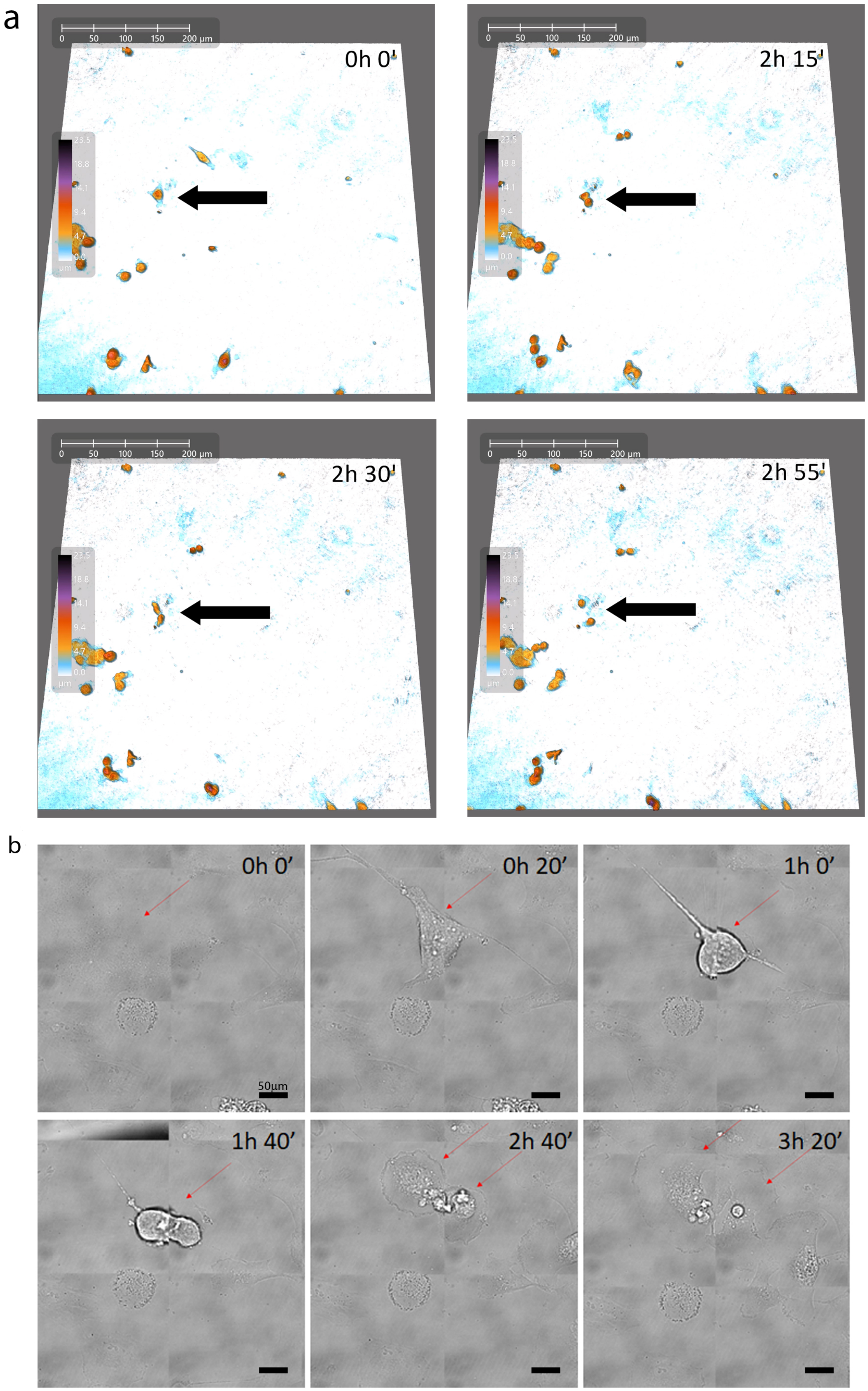

Figure S2. Analysis of division of control MDA-MB-231 cells and cells treated with dox on day D1+12. a: Snapshots of control cell division from the movies recorded with holographic microscope. b: Snapshots of asymmetric division of polyploid giant cell on day D1+12 of treatment from the movies recorded with confocal spinning disc microscope.

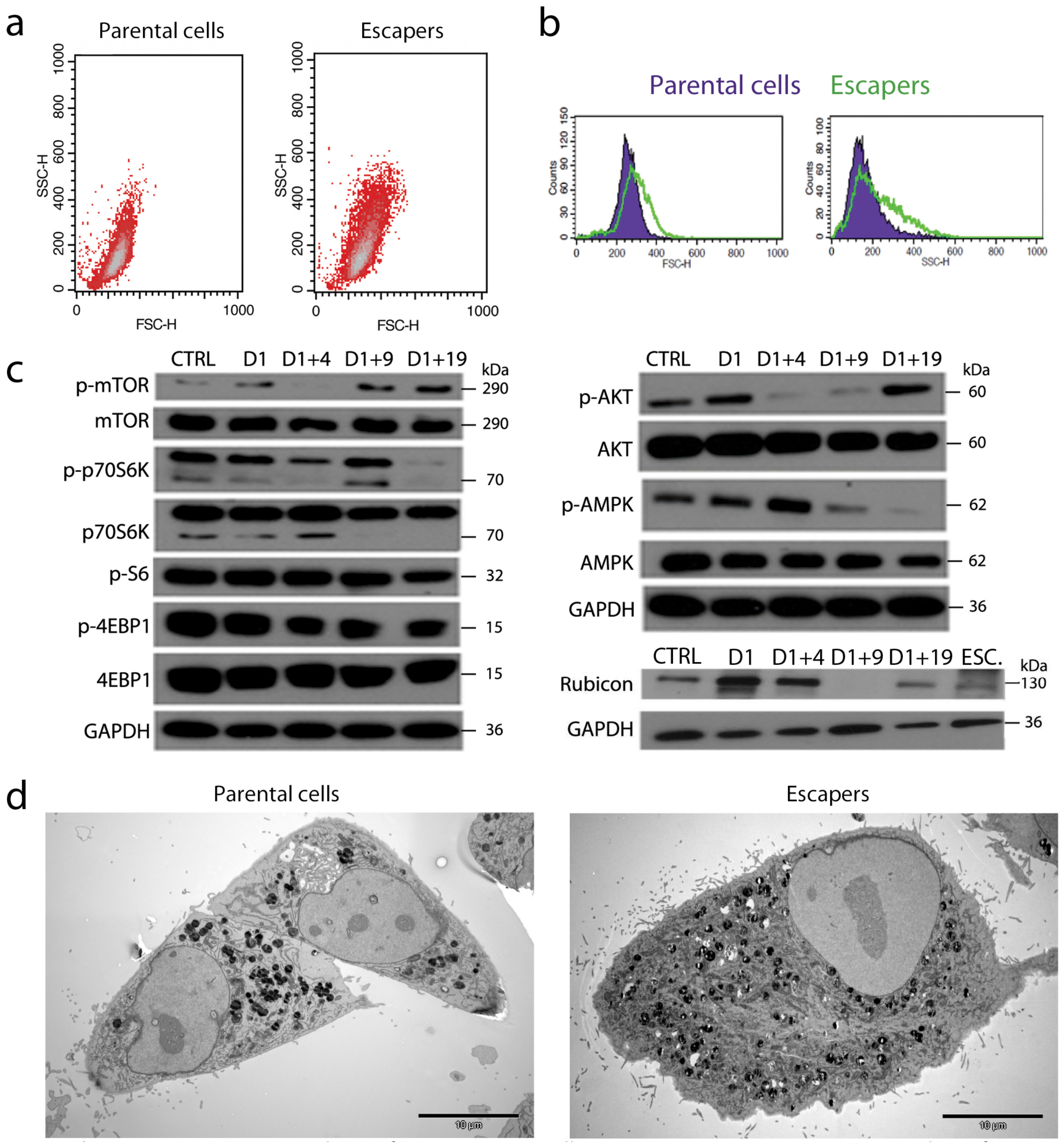

Figure S3. Analysis of MDA-MB-231 cells escaping senescence. a: Representative dot plots of size and granularity analysis by flow cytometry. b: Quantitative analysis of size and cell granularity of parental cells and escapers performed using flow cytometry. c: Representative western blots showing protein level of p-mTOR, mTOR, p-p70S6K, p70S6K, p-S6, p-4EBP1, 4EBP1, p-AKT, AKT, p-AMPK, AMPK and Rubicon. d: Representative electron microscopy images showing changes in morphology of cells.

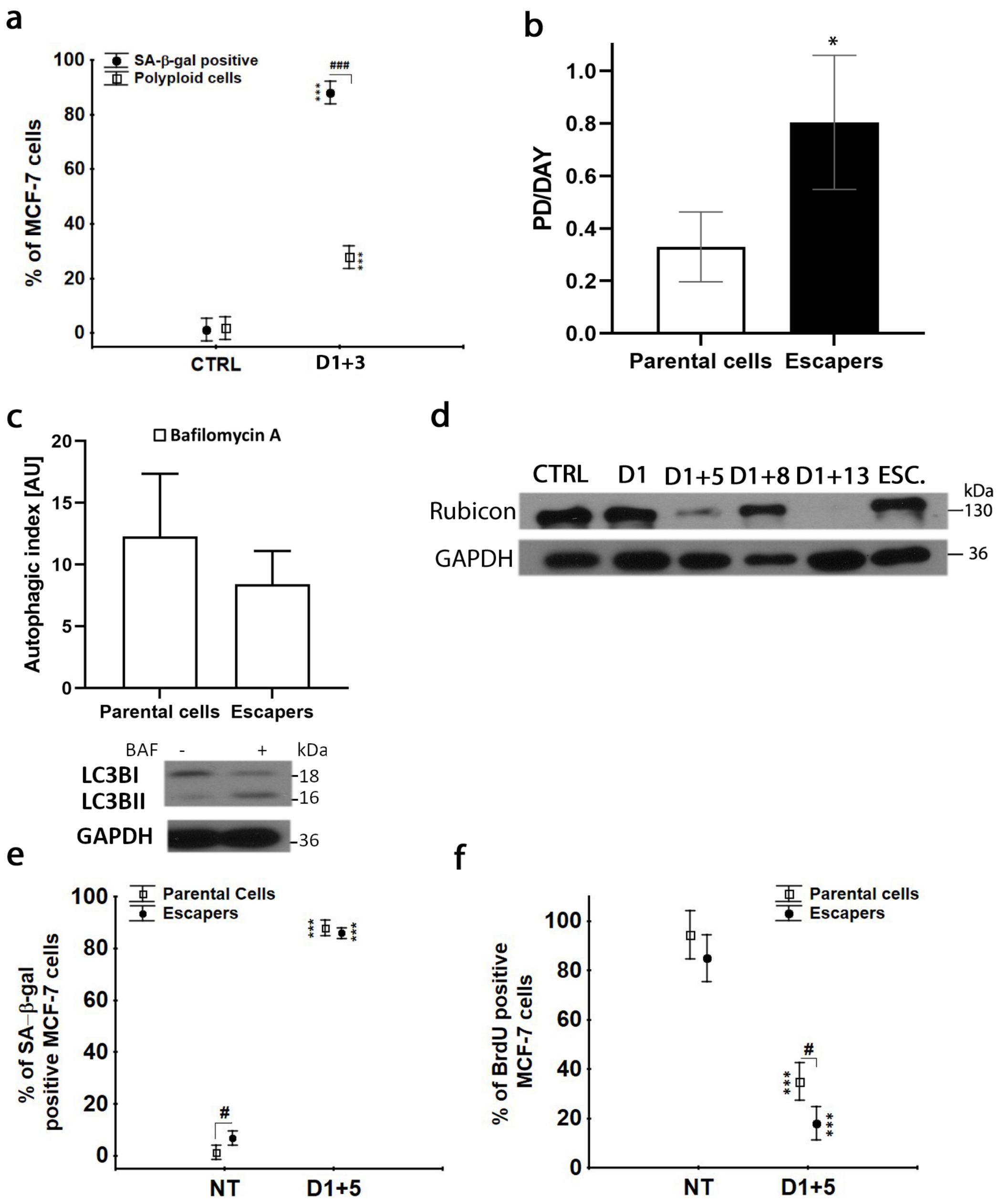

Figure S4. Analysis of MCF-7 parental cells and cells escaping senescence. a: Percentage of SA-β-gal-positive cells and polyploid ones. Data are calculated as the percentage of the total cell population, N=3 b: Analysis of population doublings per day. c: Quantitative analysis of autophagic index based on densitometry of LC3B protein level in untreated and Bafilomycin A-treated cells. d: The protein level of Rubicon; representative western blot. e-f: Percentage of SA-β-gal- and BrdU-positive cells. Data are calculated as the percentage of the total cell population. N=4 Statistics: Bars: mean value, error bars: SEM, each point: mean value ± 0.95 confidence interval. Statistical significance (in relation to non-treated (NT) or arental cells): $p < 0.05$-*, $p < 0.01$-**, $p < 0.001$-***, # - between samples.
